# Impact of Sleep Quality on Oral Surgical Complications in Dental Interns

**DOI:** 10.1111/eje.70199

**Published:** 2026-06-06

**Authors:** Tolgahan Kara, Nursena Ünlü Kuzu, Beyza Dal

**Affiliations:** ^1^ Tokat Gaziosmanpaşa University Dentistry Faculty Department of Oral and Maxillofacial Surgery Tokat Turkey

**Keywords:** anxiety, clinical skills, dental education, oral and maxillofacial surgery, sleep quality

## Abstract

**Introduction:**

Many dentistry students suffer from intense stress, insomnia, and anxiety throughout their education, especially when they start clinical practice. This study aims to evaluate the effects of sleep quality on complication rates among 5th‐grade dentistry students undergoing oral and maxillofacial surgery internships.

**Methods:**

This cross‐sectional observational study evaluated the association between sleep quality, anxiety levels, and clinical complication rates among senior dental interns during an oral and maxillofacial surgery internship. Sleep quality and anxiety were assessed using the Richard‐Campbell Sleep Quality Scale and Beck Anxiety Inventory over a 4‐week period. Clinical performance was evaluated by recording the number and types of complications occurring during routine tooth extractions. Generalized linear mixed modelling (GLMM) with Poisson distribution and log link function was used to evaluate complication incidence rates while accounting for repeated observations within students. Pearson correlation, independent samples t‐test, and Mann–Whitney U test were additionally used for statistical analysis.

**Results:**

Ninety‐two dental interns (61 female, 31 male) performed a total of 1320 tooth extractions during the study period, with 211 recorded complications. Generalized linear mixed modelling demonstrated that sleep quality was significantly associated with complication incidence rates (IRR = 0.997, *p* = 0.023). Anxiety scores were negatively correlated with sleep quality (r = −0.316, *p* = 0.002) but were not independently associated with complication incidence rates. No significant differences in sleep quality, anxiety scores, or complication rates were observed between genders.

**Conclusion:**

Poor sleep quality was associated with higher clinical complication rates among dental interns during OMFS training. Anxiety levels were negatively correlated with sleep quality but did not independently predict complication rates. Due to the cross‐sectional design and the presence of multiple uncontrolled clinical factors, these findings should be interpreted cautiously.

## 1 Introduction

The clinical internship phase in dental education is crucial for future dentists' training and professional development. This phase encompasses the final 2 years of dental education and involves practical clinical courses where complex procedures are executed and high‐risk decision‐making processes are undertaken. Consequently, this increases students' stress levels, adversely impacting their psychological and mental well‐being and clinical performance, particularly in the oral and maxillofacial surgery (OMFS) section [1, 2].

Recently, the mental health of healthcare students, especially concerning sleep quality and anxiety types, has garnered significant attention. Sleep disturbances are consistently associated with increased psychological dysfunction and cognitive disruption [3]. Among dental students, stress and insomnia are prevalent, with academic overload, patient management responsibilities, and a high volume of surgical cases contributing to these conditions [4].

Evidence indicates that poor sleep quality exacerbates anxiety symptoms, which negatively affect clinical judgement and increase the likelihood of procedural complications [5]. Research suggests that sleep disturbances among students lead to decreased clinical confidence and diminished patient safety competencies, particularly when performing invasive procedures and high‐stress clinical tasks [6]. Sleep‐related impairments reduce cognitive flexibility and significantly elevate the risk of medical errors [7]. Beyond its impact on psychological well‐being, sleep deprivation has been shown to impair vigilance, attention, reaction time, and executive functioning, all of which are critical for safe clinical practice [8]. Studies in medical and surgical settings have shown that insufficient sleep is associated with decreased technical performance, impaired decision‐making ability, and increased error rates, particularly during complex or high‐risk procedures [9]. Experimental and simulation‐based studies have shown that sleep deprivation negatively impacts fine motor skills and the accuracy of procedures, highlighting its relevance to surgical disciplines [10].

The existing literature has thoroughly examined sleep quality effects on psychological health and academic performance in dental students; yet few studies have investigated how sleep disturbances affect actual clinical results, especially during surgical internships. Prior research primarily examines self‐reported stress and academic grades instead of objective clinical procedure complication rates. The established connection between anxiety and sleep quality does not fully explain its direct effects on hands‐on clinical competency, particularly in high‐risk oral and maxillofacial surgery (OMFS) domains. The investigation of this relationship holds significance because complications impact both patient results and student educational experiences and professional growth in the future.

This study aims to explore the association between dental interns' sleep quality, anxiety levels, and clinical complication rates during oral and maxillofacial surgery internships. The null hypothesis of the study was that there would be no association between sleep quality, anxiety levels, and clinical complication rates among dental interns.

## 2 Materials and Methods

### 2.1 Study Design

This cross‐sectional study was conducted on senior students training in OMFS internship at a Faculty of Dentistry in 2024–2025. The informed consent form was obtained from all students included in the study. Ethics committee approval with number 24‐MOBAEK‐025 was obtained from the Non‐Intervention Scientific Research Ethics Committee. One of the main factors in choosing only senior dental students for the study was that fourth‐grade interns require a long adaptation and experience‐gaining process because they are exposed to the clinical environment for the first time. Another was that in the fifth‐grade internship, the last step before graduation, supervisors have relatively less influence on the student, and the intern has more control over clinical decision‐making and implementation. Richard‐Campbell Sleep Quality Scale and Beck Anxiety Inventory were used to determine the interns' sleep quality and anxiety scores. Complication counts and types were also recorded by the supervisors. All volunteer students in the OMFS internship were included in the study. Interns with long‐term absences for various reasons were excluded from the study. All clinical procedures were performed in the oral and maxillofacial surgery clinic under the direct supervision of experienced oral and maxillofacial surgery residents. Supervisors were responsible for overseeing all procedures and for recording the occurrence and type of complications.

### 2.2 Questionnaires

Within the scope of the study, the Richard‐Campbell Sleep Quality Scale was administered daily, and the Beck Anxiety Inventory was administered at the beginning of each week to the intern 5th‐grade students in the OMFS clinic.

#### 2.2.1 Richard‐Campbell Sleep Scale

The Richard‐Campbell Sleep Scale is a 6‐item scale that evaluates sleep quality based on parameters such as time to fall asleep, frequency of awakening, and ambient noise level [11]. The validity and reliability of its Turkish version were performed by Özlü&Özer [12]. Each item in this scale is assessed using the visual analog scale technique with a chart between 0 and 100. A score between “0–25” indicates severely inadequate sleep, and a score between “76–100” indicates decent sleep. The score of the 6th item, which evaluates the noise level, is excluded from the current sum of the other five items to find the total score of the item. It is considered that the higher the total score, the better the sleep quality [12].

#### 2.2.2 Beck Anxiety Inventory (BAI)

BAI is a brief measure of anxiety that consists of 21 items with a focus on somatic symptoms of anxiety [13]. The respondents were asked to indicate how much each symptom bothered them over the last one. Responses are rated on a 4‐point Likert scale and range from 0 (not at all) to 3 (severely) [14]. The higher points of this scale reflect a higher level of anxiety.

### 2.3 Recording Clinical Performances

The number of tooth extractions performed by the students and the number and types of complications encountered were recorded daily. The measurements were performed in the second half of the 2‐month internship period to minimize the effect of factors such as acclimatization to the clinical setting and experience level on the results. All extractions included in the study were routine simple extractions performed under local anaesthesia using bein elevators and forceps. Teeth requiring surgical extraction, flap elevation, or bone removal were excluded from the student clinic and referred to faculty surgeons. Preoperative radiographs were routinely used for case assessment and treatment planning. Although all procedures consisted of routine non‐surgical extractions, tooth‐related anatomical variables such as root morphology and periodontal condition were not standardized between cases.

### 2.4 Definition and Classification of Complications

Complications were classified as anticipated and preventable events. Anticipated complications included root fractures that may occur during routine forceps extractions, particularly in multirooted or periodontally compromised teeth. Preventable complications included soft tissue injury and displacement of tooth roots into anatomical spaces. All complications were recorded by supervising clinicians at the time of the procedure.

### 2.5 Statistical Analysis

Complication outcomes were analysed using generalized linear mixed modelling (GLMM) with Poisson distribution and log link function because complication data represented repeated count measurements obtained from the same students over multiple days. Daily extraction counts were included as an offset variable to account for differences in clinical exposure between observations. Student ID was included as a random intercept effect to account for within‐subject clustering. Sleep quality and anxiety scores were entered as fixed effects. Independent samples *t*‐tests, Mann–Whitney U tests, and Pearson correlation analyses were additionally performed where appropriate. Statistical analyses were conducted using IBM SPSS Statistics 26 software, and *p* < 0.05 was considered statistically significant.

## 3 Results

Ninety‐two students, 61 female and 31 male, participated in the study. Interns extracted a total of 1320 teeth during the study period. The total number of complications encountered in these tooth extractions was 211. Most complications were root fractures, accounting for the majority of observed events (Table 1).

**TABLE 1:** Distribution of complication types observed during routine tooth extractions.

Complication type	*N*	Frequency (%)
Root fracture	206	97.6
Displacement of tooth root into an anatomical area	2	0.9
Soft tissue injury	3	1.4
Total	211	100

Beck anxiety and sleep quality scores and complication rates did not significantly differ when evaluated according to gender (Table 2). The study's primary objective, namely the relationship between complication incidence rates and sleep quality and anxiety scores, was evaluated using generalized linear mixed modelling (GLMM) with Poisson distribution and log link function. Daily extraction counts were included as an offset variable, and student ID was modelled as a random intercept effect. Sleep quality was significantly associated with complication incidence rates (IRR = 0.997, 95% CI: 0.995–1.000, *p* = 0.023), whereas anxiety scores were not significantly associated with complication incidence rates (IRR = 1.015, 95% CI: 0.964–1.069, *p* = 0.569) (Table 3). Complication rates were also compared between female and male dental interns, and this comparison is illustrated in Figure 1.

**TABLE 2:** Comparison of sleep quality and anxiety scores between female and male dental interns.

	Female (*n* = 61)	Male (*n* = 31)	Test statistics	*p* *
Beck Anxiety	7,6 ± 4,8	6,4 ± 5,5	1059	0,292
Sleep Quality	403,5 ± 99,5	384,3 ± 108,3	0,851	0,397

* Values are presented as mean ± SD. Group comparisons were performed using independent samples *t*‐tests.

**TABLE 3:** Generalized linear mixed‐effects Poisson regression model examining predictors of complication incidence rates.

Predictor	Estimate (*β*)	SE	IRR	95% CI	*p*
Sleep Quality	0.003	0.001	0.997	0.995–1.000	**0.023**
Anxiety Score	0.015	0.027	1.015	0.964–1.069	0.569

*Note:* GLMM with Poisson distribution and log link function. Student ID was included as a random intercept effect to account for repeated observations within students. Daily extraction counts were included as an offset variable. Bold values indicates the value of *p* smaller than 0.05 demonstrates statistically significance.

Abbreviations: CI, confidence interval; IRR, incidence rate ratio.

**FIGURE 1:**
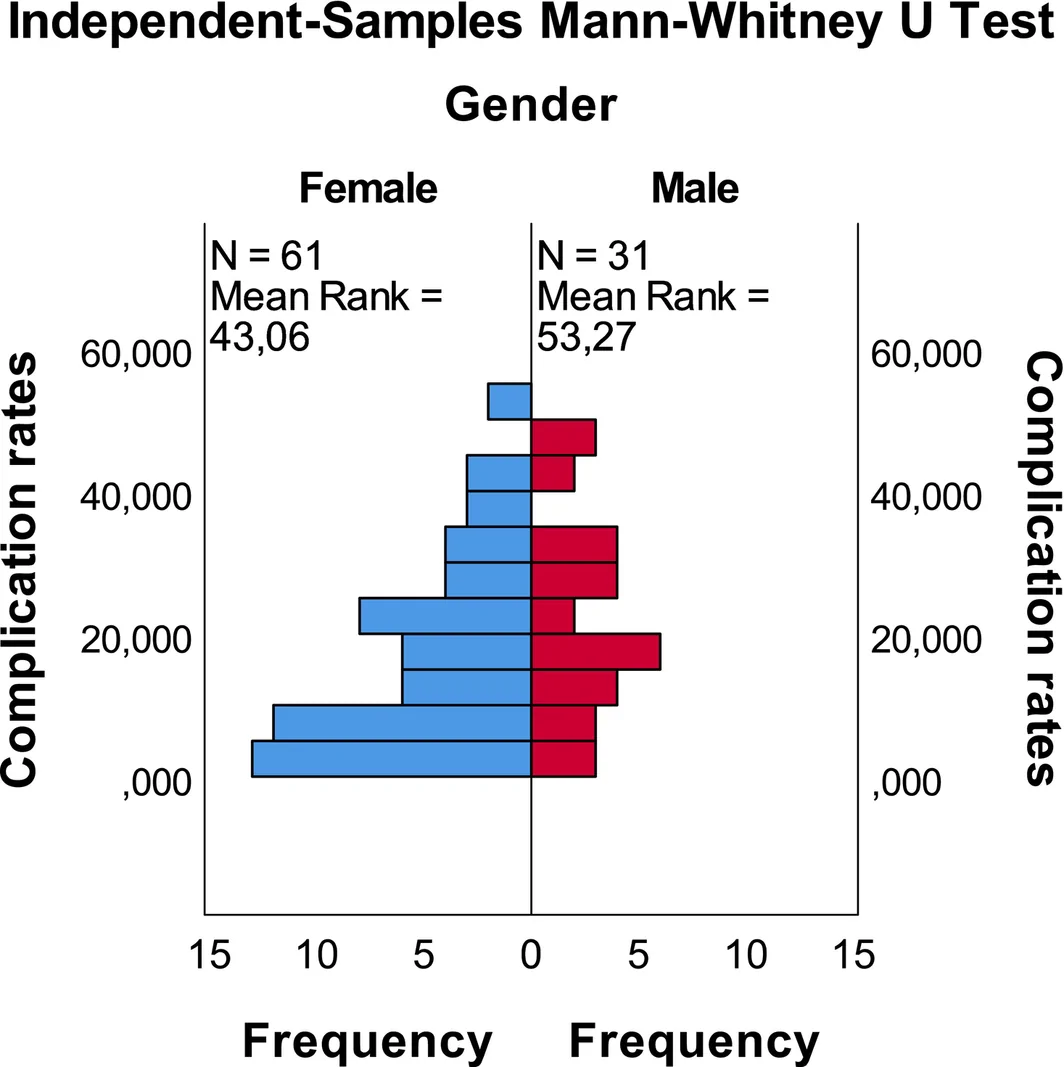
Comparison of complication rates between female and male dental interns. No statistically significant difference was observed (Mann–Whitney U test, *p* = 0.082).

Additionally, there is a significant moderate negative correlation between sleep quality and anxiety (r = −0.316, *p* = 0.002).

## 4 Discussion

The primary aim of the present study was to evaluate the effect of sleep quality levels on clinical complication rates of 5th‐grade dental interns in the OMFS section. The findings of the present study support an association between poorer sleep quality and higher complication rates. On the other hand, there is a negative correlation between an increase in anxiety and a decrease in sleep quality, and it was concluded that anxiety does not affect complication rates. In different studies, it is reported whether anxiety and poor sleep quality are the cause or result of the other. For example, Ganidagli et al. [15] concluded that anxiety was one of the risks of poor sleep quality. In another study, poor sleep quality was found to partially mediate the relationship between social anxiety and depressive symptoms in adolescents [16]. The observed relationship between anxiety, sleep quality, and complication rates suggests that these variables may interact with one another; however, the present study design does not allow determination of directional or causal relationships. An additional strength of the present study is that repeated daily clinical observations obtained from the same students were analyzed using generalized linear mixed modelling with Poisson distribution. This approach allowed within‐subject clustering to be considered while also adjusting for differences in clinical exposure using daily extraction counts as an offset variable. Accordingly, the present findings may provide a more robust evaluation of complication incidence rates during clinical dental training.

Prolonged hours of practice and intensive theoretical lessons in dental education compromise sleep quality and increase anxiety levels [17, 18]. The results reported by Angelin et al. [19] are quite remarkable. In their study, where they evaluated the sleep quality and daytime sleepiness of 1st and 2nd‐year dentistry students, they found that 71.1% had poor sleep quality and more than half (58.1%) had daytime sleepiness. Jie et al. [20] determined sleep quality and its associated factors among dental students in Malaysia. Their study found that accommodation and caffeine consumption have been significantly associated with poor sleep quality. In addition, like many studies in the literature, they also evaluated the relationship between sleep quality and the academic success of dental students and found statistically significant results. Another study found a more significant association between poor sleep quality and lower academic achievement in undergraduate dental students compared to postgraduate dental students [21]. The current study focused on the relationship between dental students' sleep quality and clinical success, not academic success. For this purpose, the tooth extraction complications they experienced in the OMFS clinic were taken as a basis. Consequently, this study contributes to the literature from a different perspective.

Since surgical procedures require precise motor skills, attention to detail, and rapid decision‐making, they are more vulnerable to the negative effects of inadequate or poor‐quality sleep. Studies using surgical simulators have found that insufficient sleep negatively affects residents' surgical skills and increases errors [22]. However, Quan et al. [23] reported no significant association between sleep deficiency and surgical errors, but they noted that sleep deficiency of surgeons might increase the operation time. Unfortunately, no studies evaluate the effect of clinicians' sleep quality on surgical practices, neither in surgical internships during dental education nor in oral maxillofacial surgery specialty training. However, Elagra et al. [17] have commented that poor sleep quality increases attention deficit and anxiety and reduces the ability to make correct decisions, negatively affecting performance, especially in the clinical practice phase of dental education. The current study contributes to the literature by examining the relationship between sleep quality, anxiety levels, and surgical complications experienced by dental students, and identifying significant associations.

Beyond dental education, a substantial body of evidence from medical and surgical training literature indicates that sleep deprivation adversely affects key components of clinical performance, including vigilance, attention, reaction time, and executive functioning [24]. These cognitive and psychomotor impairments have been associated with reduced technical efficiency, prolonged task completion times, and compromised situational awareness, particularly in high‐risk or procedurally demanding clinical settings [25]. Importantly, several studies emphasize that the impact of sleep deprivation may manifest not only as overt errors but also as subtle declines in performance quality that are more difficult to capture using binary error‐based outcomes [10]. In this context, the present findings may be interpreted as contributing to the growing evidence that sleep quality represents a relevant factor in clinical performance, even during routine procedures performed under supervision.

A significant correlation between sleep quality and anxiety, the secondary result of our study, is also consistent with the literature. Sanusi et al. [26] also reported a significantly moderate correlation between perceived stress and sleep quality. Similarly, a study about self‐reported bruxism in dental students noted a significantly weak correlation between sleep quality and anxiety [27]. Even though some studies showed adverse effects of anxiety on communication skills, diagnostic skills, and psychomotor performance [28, 29, 30], Lugassy et al. [31] found that DASS scores (including depression, anxiety, and stress) of dental students did not correlate with clinical performances. The current study showed that anxiety did not affect students' clinical performance, similar to Lugassys' outcomes. However, the absence of a statistically significant association between anxiety and complication rates should be interpreted with caution. Given the borderline nature of the statistical findings, the possibility of a Type II error cannot be excluded, and insufficient statistical power may have limited the detection of a true association.

This study has some limitations. First, it was conducted in a single center with a small sample size. This reduces the generalizability of the results to the general dental student population. Another is that the measurement of sleep quality and anxiety levels was based on the participants' own reports. The vast majority of recorded complications consisted of root fractures. Root fracture during tooth extraction is influenced by multiple tooth‐ and patient‐related factors, including root morphology, tooth location, degree of caries destruction, periodontal support, patient age, and bone density. These variables were not standardized or controlled in the current study and may have contributed substantially to complication occurrence independently of sleep quality. Therefore, the observed associations should not be interpreted as evidence that sleep quality alone determines clinical complication rates. Although repeated daily observations were analyzed using mixed‐effects modelling, extraction‐specific data were unavailable; therefore, clustering at the individual extraction level could not be directly evaluated. Future studies should be conducted in multiple centers with larger student populations and using different measurement and assessment methods.

## 5 Conclusion

Within the limitations of this cross‐sectional study, poorer sleep quality was associated with higher clinical complication rates among senior dental interns during OMFS training. Anxiety levels were negatively correlated with sleep quality but were not independently associated with complication rates. Since multiple procedural and patient‐related factors may influence extraction complications, the findings should be interpreted cautiously and do not establish a causal relationship. Further multicenter prospective studies with standardized clinical variables and objective sleep assessments are needed to better clarify these associations.

## Funding

The authors have nothing to report.

## Ethics Statement

Ethics committee approval with number 24‐MOBAEK‐062 was obtained from Tokat Gaziosmanpasa University Non‐Intervention Scientific Research Ethics Committee.

## Conflicts of Interest

The authors declare no conflicts of interest.

## Data Availability

The data that support the findings of this study are available from the corresponding author upon reasonable request.
